# The Need for Standardization in Next-Generation Sequencing Studies for Classic Hodgkin Lymphoma: A Systematic Review

**DOI:** 10.3390/diagnostics12040963

**Published:** 2022-04-12

**Authors:** Antonio Santisteban-Espejo, Irene Bernal-Florindo, Jose Perez-Requena, Lidia Atienza-Cuevas, Julia Moran-Sanchez, María del Carmen Fernandez-Valle, Raquel Romero-Garcia, Marcial Garcia-Rojo

**Affiliations:** 1 Department of Pathology, Puerta del Mar University Hospital, 11009 Cadiz, Spain; antoniosantistebanespejo@gmail.com (A.S.-E.); jose.perez.sspa@juntadeandalucia.es (J.P.-R.); lidia.atienza.sspa@juntadeandalucia.es (L.A.-C.); marcial.garcia.sspa@juntadeandalucia.es (M.G.-R.); 2 Institute of Research and Innovation in Biomedical Sciences of the Province of Cadiz (INiBICA), 11009 Cadiz, Spain; raquel.romero.garcia@juntadeandalucia.es; 3 Department of Medicine, Faculty of Medicine, University of Cadiz, 11003 Cadiz, Spain; juliamorsan@gmail.com; 4 Department of Hematology and Hemotherapy, Puerta del Mar University Hospital, 11009 Cadiz, Spain; macafeva@hotmail.com

**Keywords:** Classic Hodgkin lymphoma, standardization, next-generation sequencing, liquid biopsy

## Abstract

Classic Hodgkin lymphoma (cHL) constitutes a B cell-derived neoplasm defined by a scarce tumoral population, termed Hodgkin and Reed–Sternberg (HRS) cells, submerged into a histologically heterogeneous microenvironment. The paucity of HRS cells has historically hampered genetic studies, rendering the identification of the recurrent genetic lesions and molecular pathways deregulated in this lymphoma difficult. The advent of high-throughput sequencing methods such as next-generation sequencing (NGS) could sensibly optimize the identification of the mutational landscape of cHL. However, there is no current consensus either in the design of panels for targeted NGS or in its most relevant clinical applications. In this work, we systematically review the current state of NGS studies of cHL, stressing the need for standardization both in the candidate genes to be analyzed and the bioinformatic pipelines. As different institutions have developed and implemented their own customized NGS-based protocols, to compare and systematically review the major findings of this ongoing research area could be of added value for centers that routinely perform diagnostic, monitoring and genotyping strategies in cHL samples. The results of this systematic review should contribute to the interdepartmental harmonization and achievement of a consensus in the current clinical applications of NGS studies of cHL.

## 1. Introduction

Classic Hodgkin lymphoma (cHL) constitutes a B cell neoplasm derived from germinal center B cells at different stages of development. From its early description [1] and cytological characterization [2,3], the lineage assessment of cHL has proved to be a major challenge because the tumoral fraction, termed Hodgkin and Reed–Sternberg (HRS) cells, constitutes a scattered population (1–5%) surrounded by a dominant microenvironment consisting of B and T lymphocytes, macrophages, eosinophils, histiocytes and plasma cells [4]. Micromanipulation and single-cell PCR allowed the identification of the B cell origin of HRS cells [5].

Both basic and translational research have allowed the genomic profiling of cHL [6] and the identification of the constitutive activation of the JAK/STAT [7,8,9] and NF-kB [10,11] signaling pathways, revealing a highly organized mechanism of immune evasion via the amplification of the PDL (programed death ligand) genes localized in 9p24.1 [12,13].

The advent of high-throughput DNA sequencing methods such as next-generation sequencing (NGS) could sensibly improve the diagnostic, risk-stratification and follow-up procedures for patients diagnosed with cHL. To this end, the achievement of an international consensus on the design of personalized or targeted NGS panels constitutes a fundamental issue. Different laboratories have used their own protocols and, consequently, the literature in this area is heterogeneous. Recently, the need for a consensus in NGS studies for mature B cell neoplasms has been highlighted by the French LYSA (Lymphoma Study Association) and GBMHM (Groupe de Biologistes Molécolares des Hemopathies Malignes) [14].

The early recognition of the subgroup of cHL patients with refractory or relapsed disease (high-risk cHL), which constitutes about 10–20% when using Adriamycin-based protocols [15], requires a unifying approach for the molecular assessment of risk. Due to the difficulty in accessing a sufficient DNA concentration in the scarce tumoral population of biopsied tissues and to minimize the side effects of radiation, circulating tumor DNA (ctDNA) coupled with NGS has emerged as a promising strategy for the diagnosis and monitoring of patients with cHL.

To the best of our knowledge, the current state of the main clinical applications of NGS of cHL has not been previously systematically reviewed. The main aim of this work is to gather and analyze all potentially relevant publications evaluating the clinical application of NGS in the management of cHL, exposing the characteristics of each one as well as their advantages and limitations. The reported findings should contribute to the standardization and achievement of a consensus in the design and validation of NGS analyses in this entity.

## 2. Materials and Methods

### 2.1. Search Strategy

This systematic review was conducted and reported following the Preferred Reporting Items for Systematic Reviews and Meta-Analyses (PRISMA) guidelines on systematic reviews [16]. Before the start of the search, a review protocol was entered into the PROSPERO database (320059).

The literature search was conducted in the period of January–February 2022 in the following electronic databases: the Clarivate Analytics Web of Science (WoS) database of the Thomson Reuters Institute for Scientific Information (ISI) (Philadelphia, PA, USA) and MEDLINE (National Library of Medicine, Bethesda, MD, USA) through the PubMed interface.

Combinations of different search terms with Boolean operators (AND, OR) were used for retrieving all potentially relevant documents. The literature search was restricted to articles published in the previous five years (2017–2022). The search terms and the number of records obtained for each database are shown in Table 1. Research conducted on human samples and published in the English language were considered to be the filters.

### 2.2. Selection Criteria

The inclusion and exclusion criteria were previously defined to consider the eligible documents. Publications were included if they met one of the following inclusion criteria: (1) documents specifically addressing the clinical usefulness of NGS in the study of cHL; (2) documents evaluating different techniques of molecular biology in cHL samples including NGS; and (3) publications applying NGS for the study of different lymphoid neoplasms including cHL samples. Documents reporting the results of molecular assays other than NGS in lymphoid neoplasms or without including cHL tumor samples were not included in this systematic review.

### 2.3. Study Selection Process and Data Extraction

The literature search was carried out by combining the search terms indicated above. Duplicated articles were removed. The titles and abstracts were then reviewed and those articles that did not meet the inclusion criteria were excluded. The remaining articles were independently analyzed by three reviewers (A.S.-E., I.B.-F. and J.P.-R.). Subsequently, the conflicting registries were discussed by the three initial reviewers and an additional fourth reviewer (M.G.-R.) to reach a consensus.

The list of bibliographic references of the documents screened was also reviewed to capture additional relevant publications. Articles selected in this process were considered to be additional records identified through other sources. Finally, after full-text reading, the data extracted from each publication were: (1) authors and year of publication; (2) goal of the NGS experiment; (3) sample size and clinical features of the samples studied (diagnostic or relapsed/refractory samples); (4) origin of the tumoral DNA; (5) sequencing chemistry used; (6) bioinformatic pipeline employed; (7) major findings of the study; and, finally, (8) potential clinical (diagnostic, prognostic and therapeutical) applications.

## 3. Results

**Figure 1:**
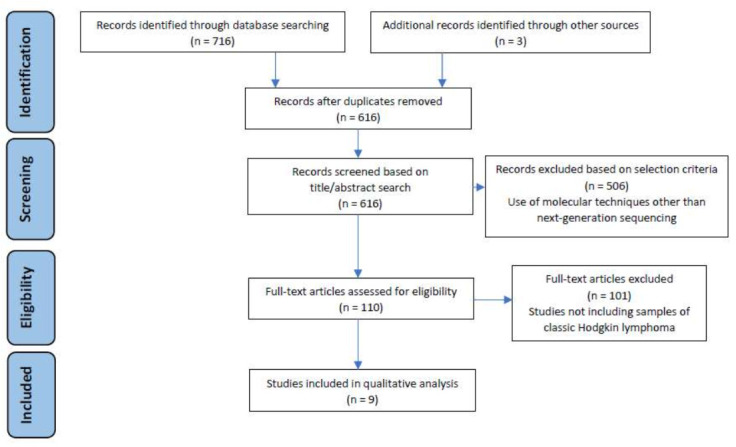
Study selection flow diagram. The literature search was performed in the period January–February 2022 on Web of Science and MEDLINE databases, following the PRISMA guidelines [16].

The search strategy conducted led to the identification of 716 possibly relevant publications. After the duplicates were removed, 616 documents were submitted for title/abstract screening. A total of 506 articles were discarded based on the exclusion criteria (i.e., they reported the use of molecular techniques other than NGS). After full-text accessing, 101 articles were withdrawn because they did not specifically report the results of NGS on cHL or they used NGS technologies on different lymphomas without including tumor samples of cHL. Finally, 9 articles were eligible for the critical review and qualitative synthesis (Figure 1).

Of note, after the analysis and data extraction from these nine documents, three major applications of NGS in the study of cHL could be distinguished: (1) the assessment of clonality through the identification of immunoglobulin (Ig) gene rearrangements; (2) the analysis of ctDNA from a liquid biopsy for genotyping and patient follow-up; and (3) the molecular profiling of refractory/relapsed cHL patients aiming to personalize therapeutical decisions. The main findings of the publications analyzed in this systematic review are shown in Table 2.

### 3.1. Next-Generation Sequencing for the Assessment of Clonality in Classic Hodgkin Lymphoma

The detection of clonality could constitute an important feature in cases of cHL with diagnostic difficulties. Routine methods for the identification of Ig gene rearrangements are based on a conventional PCR (i.e., BIOMED-2/EuroClonality]. In the work by van Bladel et al. [19] a BIOMED-2/EuroClonality assay was specifically compared with NGS for clonality assessment of 16 primary node biopsies of cHL, both in whole-tissue specimens (FFPE and fresh frozen tissues) and isolated HRS cells through laser microdissection.

The conventional BIOMED-2/EuroClonality assay showed an inferior performance when compared with NGS. Clonal Ig gene rearrangements were detected in 10 out of 16 fresh frozen samples (63%) and in 3 out 15 FFPE samples (20%) by a conventional multiplex PCR. On the other hand, NGS allowed the identification of clonality in 14 out of 16 fresh frozen samples (88%) and 9 out of 16 FFPE samples (56%). Of note, the number of non-interpretable results was equal for both techniques and concordant with the type of specimen analyzed. All fresh frozen tissue specimens yielded interpretable results whereas 2 out of 16 FFPE samples yielded non-interpretable results using either the BIOMED-2/EuroClonality assay or NGS-based methods.

An advantage of an NGS-based clonality assessment is the availability of information regarding different clonotypes in the same tumoral specimen. Clonal diversity has been evidenced by showing multiple unrelated clonal Ig gene rearrangements in the same cHL cases (such as combined unproductive IGHD-IGHJ gene rearrangements and productive IGHV-IGHD-IGHJ gene rearrangements) or even involving both IGH and IGK genes [19].

In addition, deep sequencing allows for the recognition of clonality in composite and sequential lymphomas (i.e., the coexistence of cHL and follicular lymphoma) in which the identification of different clonal trajectories could be of diagnostic and prognostic importance. Identical clonotypes were identified through NGS-based IGK gene rearrangements in three cases of composite cHL–follicular lymphomas [17]. Of note, cell compartments showed different mutational patterns with XPO1 (cHL compartment), FOXO1 and TNFRSF14 (follicular compartment) being the most frequently mutated genes.

Considering the key prognostic role of the different cell populations integrating the tumoral microenvironment in cHL such as cytotoxic T lymphocytes [26], T regulatory (Treg) lymphocytes [27] and macrophages [28,29,30], this technical advantage of an NGS-based assessment of clonality could be of major importance in this disease.

### 3.2. Next-Generation Sequencing and Liquid Biopsy: New Approaches for the Diagnosis and Follow-Up of Patients with Classic Hodgkin Lymphoma

Due to the scarcity of HRS cells in tumor tissues, obtaining ctDNA from plasma samples constitutes a decisive advance for the diagnosis, genotyping and monitoring of the therapeutic response in patients with cHL. In the work by Spina et al. [25], the authors retrospectively analyzed ctDNA from 80 newly diagnosed and 32 refractory or relapsed cHL patients by using a customized panel of 77 genes recurrently mutated in mature B cell neoplasms (see Supplementary Material Table S1).

Of note, the plasma samples were collected at different timepoints during the course of the treatment, allowing longitudinal comparisons: at diagnosis (n = 80); under chemotherapy on the first day of the second cycle (before the interim PET) (n = 24); after progression (n = 32); and before and after failing autologous hematopoietic transplantation (n = 6), brentuximab–vedotin (n = 6) and nivolumab (n = 5). Mutations identified in the ctDNA and purified HRS cells in the biopsies were highly concordant (87.50%; 95% confidence interval: 79.20–92.80%), including variants of the TNFAIP3, ITPKB, GNA13 and B2M genes, which have been previously reported in studies of isolated HRS cells [31,32]. Furthermore, the pretreatment ctDNA concentration correlated with the Ann Arbor stage (*p* = 0.021), limited and advanced stages (*p* = 0.001) and German Hodgkin Study Group (GHSG) risk model (*p* = 0.013), also confirming that ctDNA constitutes a surrogate biomarker of the tumor load. When ctDNA was analyzed in refractory cHL patients, different clonal evolution trajectories could be identified, depending on the treatment modalities. In patients relapsing after chemotherapy and brentuximab–vedotin, selective pressure induced by the drugs only partially modified the genotype of the HRS cells, leaving the ancestral clones almost intact. However, the achievement of periodical remissions under nivolumab courses was accompanied by the emergence of new clones, reflecting the acquisition of new mutations in response to immunotherapy.

A formal prospective evaluation of the value of ctDNA in cHL management was conducted by Camus et al. [20] (NCT: 02815137). In a cohort of 60 patients, a reduced 9-gene customized NGS panel was employed at diagnosis and after C2, showing that the most frequent variants involved the cytokine-regulator gene SOCS1 (50%). Concentrations of ctDNA correlated with the presenting features and metabolic tumor volume (MTV) (ρ = 0.57; *p* < 0.001), but no statistically significant differences were observed between the ctDNA levels for Deauville Score (DS) 1–3 patients compared with DS 4–5 patients (*p* = 0.79). From the 147 genetic variants identified, a paired analysis (biopsies and plasma samples) showed an acceptable concordance (Cohen’s κ = 0.56 (range: 0.23–0.89)), indicating that 61/147 variants identified were obtained both in the biopsy and plasma of the same patient.

In pediatric HL (PHL), Desch et al. [24] originally showed that ctDNA is also a feasible origin of DNA for genotyping. In 96 pediatric patients (average age: 14 years; range: 3–18) enrolled in the EuroNet-PHL-C2 trial [23], recurrent mutations were found in the members of the JAK/STAT and NF-kB signaling pathways. The most frequently mutated genes were SOCS1 (80%), IGLL5 (33%) and TNFAIP3 (32%). As previously reported in adult cHL [20], the pretreatment ctDNA correlated with the MTV, confirming that ctDNA is a feasible source of DNA for the follow-up of PHL patients, where the minimization of radiation doses is especially important.

### 3.3. Identification of High-Risk Mutational Profiles in Classic Hodgkin Lymphoma through Next-Generation Sequencing Methods

A fraction between 20% and 30% of patients with cHL is either primary refractory or relapse after achieving a complete metabolic response. If first-line therapy fails, high doses of chemotherapy and autologous hematopoietic stem cell transplantation are only curative in half of the patients [33]. Consequently, the early recognition of high-risk cHL patients remains a major research goal.

The application of NGS to identify the recurrently mutated genes in these patients in order to propose more intensive therapies constitutes a fundamental application of these platforms. NGS could also be of added value in this setting to investigate the pathogenetic mechanisms involved in lymphomagenesis in cHL. The prognostic scores clinically validated for cHL management such as the International Prognostic Score (IPS) [34], the GHSG score [35] and the European Organization for Research and Treatment of Cancer (EORTC) system [36] are based solely on the clinical and analytical variables; a genetics-based risk model is still lacking for this disease.

The work by Mata et al. [6] studied 57 FFPE samples of cHL with NGS technologies. A total of 34% of these patients had primary refractory cHL. Single-nucleotide variants (SNVs) were identified in 23 out of the 57 cases (40.35%), but the genomic data were not categorized according to the response to therapy. The most frequently mutated genes were EP300 (12.28%), CSF2RB (12.28%), STAT6 (10.53%) and BTK (10.53%). Furthermore, treatment with BTK inhibitors decreased the proliferation rate and induced cell death in cHL-derived cell lines, suggesting the need for BCR signaling in the HRS compartment. These results reinforce the importance of the JAK/STAT and BTK pathways as well as the epigenetic modulators in the pathogenesis of cHL, as previously noted by other authors [32,37].

The same group published a paper analyzing only cases of refractory cHL with NGS studies [21]. Mutations in EP300 (41.67%), CREBBP (33.33%) and TP53 (25%) were overrepresented in this series of refractory cases. Sequencing studies were performed on the original pretreatment biopsy and the relapse biopsy, giving consistency to the data obtained.

It is of particular note that a few mutations in this study were identified in the CD30-negative cellular fraction of the node biopsies, suggesting the existence of a clonally related population. In this non-tumoral fraction, the most frequent mutations involved the chromatin-remodeling genes CREBBP (16.67%) and SMARCA4 (16.67%), coherent with the central role of epigenetic modifications in the pathogenesis of refractory cHL.

## 4. Discussion

The normal cellular counterpart of cHL has been elusive [38] until single-cell PCR studies were performed on isolated HRS cells via micromanipulation [5,39]. The scarce tumoral HRS cells present in the node biopsies of cHL patients derived from a B cell population that surpassed the physiologic apoptotic mechanisms within the GC of the lymph node [40]. Immunophenotyping of HRS cells evidenced that the tumoral fraction constitutes a paradigm of genotype–phenotype discordance [41,42] because B cell markers (CD19, CD20, CD79a and surface Ig) are usually not expressed or expressed in lower levels than normal B lymphocytes. Underlying mechanisms include crippling mutations in the promotor region of the Ig gene [43], epigenetic silencing [44,45] and the downregulation of B cell transcription factors [46]. Paradoxically, the survival of HRS cells is guaranteed even in the absence of a basic prerogative for B cell development such as the expression of surface Ig. The constitutive activation of the JAK/STAT [7,8,9] and NF-kB [10,11] molecular pathways and immune evasion via copy number gains in the PDL1 and PDL2 [12,13] loci explain, in part, the acquisition of the proliferative advantage in a defective B cell.

Although advances in the molecular pathology of cHL have allowed the development of new diagnostic and therapeutic procedures, the recent application of NGS techniques in research and clinical institutions requires harmonization as well as the achievement of a consensus in several key aspects of this disease. The main aim of this work was to systematically review the current clinical applications of NGS in the study of cHL. To this end, MEDLINE and WoS databases were consulted in the period January–February 2022 and the meaningful information was extracted, including the type of the samples evaluated and the clinical context (diagnostic, refractory or relapsed), the sequencing chemistry applied, the bioinformatic tools employed and the major findings obtained with a potential clinical impact.

NGS has been applied to cHL to assess the B cell origin of HRS cells. The recognition of the presence of Ig gene rearrangements in the neoplastic compartment of a large cell lymphoma could be of major importance, especially in cases that offer diagnostic difficulties. Anaplastic large cell lymphoma (ALCL), anaplastic lymphoma kinase (ALK)-positive is a T cell lymphoma consisting of large cells with an abundant cytoplasm and multiple nuclei that may resemble HRS cells (Hodgkin-like pattern). The immunohistochemical expression of CD30 is strong in both tumor cells with a common cell membrane and Golgi region pattern [47]. Although the differential staining of the epithelial membrane antigen (EMA) and a weak PAX5 expression could serve to establish a differential diagnosis, HRS cells contain Ig gene rearrangements in more than 98% of cases [47] whereas ALCL, ALK-positive show clonal rearrangements of the T cell receptor (TCR) genes in approximately 90% of the cases [48].

In most laboratories, the assessment of clonality is based on a conventional multiple PCR (i.e., a BIOMED-2/EuroClonality assay). However, NGS-based approaches are increasingly applied to this end and protocols specifically designed for clonality assessments of lymphoid neoplasms have been developed [49]. In cHL, the EuroClonality-NGS Working Group protocol has shown a superior performance when compared with conventional BIOMED-2/EuroClonality in the identification of Ig gene rearrangements, both in FFPE and fresh frozen samples [19]. Of note, the performance of the NGS-IG gene analysis of clonality seems to be more evident in non-Hodgkin lymphomas (NHLs) in which higher rates of clonality detection have been achieved in comparison with cHL [49]. The lower DNA concentration obtained from the scattered tumoral population in cHL could explain these differences. However, an NGS-based assessment of clonality in cHL is of little value to discriminate between a large B cell lymphoma and cHL; and composite cases (i.e., the coexistence of cHL and follicular lymphoma) are very infrequent. Consequently, both in NHL and cHL, larger studies are needed to establish clear recommendations regarding the added value of NGS in the assessment of the status of the Ig gene.

On the other hand, clonal diversity and intra-tumoral heterogeneity can be assessed with NGS-based methods, which is of special importance for detecting subclonal evolution [50] as well as for the diagnosis of sequential and composite lymphomas. The coexistence of follicular lymphoma and cHL [17] as well as plasmablastic lymphoma and cHL [51] have been previously described. Furthermore, the traditionally termed “grey zone” lymphomas are still not well-understood at the genomic level. The identification of a clonal relationship between the histologically different components of the WHO-defined category of “B cell lymphomas unclassifiable, with intermediate features between diffused large B cell lymphoma and cHL” [47] is another application of an NGS-IG gene analysis in this diagnostic setting.

So far, the scarcity of HRS cells in biopsy specimens has limited the elucidation of the genetic landscape of cHL. The possibility of analyzing ctDNA in plasma samples from cHL patients offers an opportunity to improve diagnosis as well as the genotyping and monitoring strategies of this entity. Recurrent genetic variants involving the JAK/STAT and NF-kB signaling pathways and the B2M gene have been described using DNA from fixed tissues [31,32] and plasma samples [18,20,24,25]. In one study analyzing cHL samples [20], the median variant allelic frequencies (VAF) were significantly superior in ctDNA than in a biopsy (1.99% vs. 1.6%, *p* = 0.024).

Of the nine studies analyzed in the present systematic review, four of them indicated the VAF used [17,18,20,24]. In three of them [18,20,24], the VAF cutoff was 0.5% and in another [17], it was 1%. Given the low tumor cellularity present in cHL samples and the difficulty in accessing sufficient DNA, it seems appropriate to consider a VAF equal to or greater than 0.5% for NGS studies of this entity.

The lack of standardization in the design of NGS panels could explain, in part, the differences observed among the studies. In a previous work [20], variants in the SOCS1 gene were the most frequent genomic alteration evidenced in HRS cells; another work did not report mutations in this gene because SOCS1 was not included in the customized panel [25]. A harmonization in NGS analyses of mature lymphoid neoplasms has been stressed in a previous document [14] and future liquid biopsy-based strategies require a more unified approach in order to achieve consistent results among different laboratories. There are widely used NGS panels for the study of solid tumors such as the Oncomine^®^ Focus Assay (OFA) panel (Thermo Fisher Scientific, Austin, TX, USA), but the sequencing of cHL requires a customized panel targeting the recurrent mutations in this lymphoma.

A consensus NGS panel for cHL would improve its adoption and clinical implementation in different laboratories. Regarding this, we have proposed a minimal set of genes to perform NGS studies of cHL samples (Supplementary Material Table S2), which could be of interest for centers performing genotyping analyses of cHL.

When ctDNA is used for the early recognition of patients achieving durable complete remissions or to anticipate intensification or de-escalation strategies, there is also the need to standardize the cutoff values of the ctDNA employed. A 2-log drop in ctDNA after two chemotherapy courses has been identified as the best cutoff to predict progression-free survival in cHL patients (*p* < 0.001) [25], but the retrospective design of this study limits the generalization of this finding. In fact, in the only study retrieved that prospectively evaluated ctDNA in cHL, no statistically significant association was observed between the concentration of ctDNA and the Deauville Score determined by PET [20].

As 20–30% of patients with cHL are chemorefractory or relapse after achieving a first complete remission, the mutational profiling of this high-risk subgroup constitutes a major research goal. The impossibility of prospectively comparing the genomic profiles of high-risk patients with the same cohort of “good responders” (low-risk patients) imposes a fundamental barrier.

Nevertheless, studies conducted retrospectively have shown that samples from refractory and relapsed cHL patients are enriched in mutations in the epigenetic regulators and p53 [21]. Variants in the histone acetylation domains of the EP300 (N1776H) and CREBBP (P1083L) genes were reported to be frequently mutated in this subset of patients. In contrast with previous studies that reported no mutations in TP53 [32], deep sequencing studies [22] are increasingly demonstrating that the deregulation of the p53 pathway is common in chemorefractory cHL patients, as it is well-established in other cancers.

In conclusion, the recent literature on the clinical applications of NGS studies of cHL points to the need of standardization. Liquid biopsies for the diagnosis and monitoring of the therapeutic response, clonality assessments in cases with a histological atypical presentation and the early identification of high-risk patients seem to constitute the most developed areas of ongoing research. The development of clinical trials in which the results of NGS studies are used prospectively and the accumulation of further evidence in the form of systematic reviews will allow a meta-analysis and the achievement of an international consensus in this field.

## Figures and Tables

**Table 1:** Search strategies and results for each database.

Results	Database	Search Terms
434	Web of Science	“Hodgkin lymphoma”, “Hodgkin disease”, “Next Generation Sequencing”, “NGS” and “Molecular biology”
282	MEDLINE	

**Table 2:** Main characteristics of the studies included in the present systematic review.

Clinical Usefulness	Major Findings	Bioinformatic Analysis	Sequencing Chemistry	Origin of Tumor DNA	Sample Size and Clinical Features	Goal of the NGS Experiment	Study
Adjusted chemotherapy depending on the mutational profile	ARID1A and KTM2D commonly mutated in FL and cHL. There is a secondary clonal evolution after transdifferentiation	LymphoTrack and Vidijil software	Illumina, San Diego, CA, USA	FFPE and fresh frozen tissue	3 sequential lymphomas for clonality and 5 cases for targeted NGS	To evaluate transdifferentiation between cHL and follicular lymphoma (FL)	Trecourt et al., (2021) [17]
ctDNA as a feasible strategy for genotyping and monitoring	Variants in SOCS1 (28%), IGLL5 (36%), TNFAIP3 (23%), GNA13 (23%) and STAT6 (21%). Poor prognosis features correlated with ctDNA concentration	VarScan2 and DGCaller algorithms for variant calling. RefSeq database for functional annotation	Illumina, San Diego, CA, USA	ctDNA	60 cases of newly diagnosed cHL	Identify cHL somatic variants	Alcoceba et al., (2021) [18]
NGS as a sensitive and specific assay for clonality analysis	Clonality detection rates: fresh frozen: NGS (88%) vs. BIOMED-2 (63%); FFPE tissue: NGS (56%) vs. BIOMED-2 (20%)	ARResT/Interrogate pipeline	Ion Torrent^TM^, Thermo Fisher, Waltham, MA, USA	FFPE and fresh frozen tissue	Duplicated analysis (PCR and NGS) of 16 primary cHL cases	Compare NGS and BIOMED-2/EuroClonality for IG gene rearrangement	Van Bladel et al., (2021) [19]
ctDNA as a valid tool for genotyping and response assessment	Variants in SOCS1 (50%), B2M (33.3%), TNFAIP3 (31.7%), STAT6 (23.3%) and ITPKB (23.3%). ctDNA concentration correlated with metabolic tumor volume (MTV)	Software builder for base calling, alignment and quality control (Torrent Suite)	Ion Torrent^TM^, Thermo Fisher, Waltham, MA, USA	ctDNA	60 cases of newly diagnosed cHL	Evaluate liquid biopsy as a new strategy for diagnosis and tailored treatment	Camus et al., (2021) [20]
Drugs targeting epigenetic modulators could be of interest in refractory cHL	Frequent mutations in epigenetic regulators as EP300 (41.6%) and CREBBP (33.3%)	Torrent Suite, Integrative Genomics Viewer (IGV) and PROVEAN and CONDEL algorithms	Ion Torrent^TM^, Thermo Fisher Scientific, NY, USA	FFPE	12 cHL refractory patients (paired samples from diagnosis and relapse)	Identify genomic variants in refractory cHL	Mata et al., (2019) [21]
Modifications in the variant allele frequency of XPO1 in ctDNA correlates with clinical outcomes	Variants in TP53 (22%), B2M (22%), XPO1 (18%), TNFAIP3 (14%) and SOCS1 (10%) in biopsied tissues. XPO1 was detected in 31% of ctDNA	Not detailed	Illumina, San Diego, CA, USA	FFPE tissue and ctDNA	63 cHL patients (clinical features not specified)	Describe the mutational profile of cHL by CGP	Liang et al., (2019) [22]
ctDNA quantification as a useful tool for monitoring pediatric HL patients	SOCS1 (80%), IGLL5 (33%) and TNFAIP3 (32%). Pretherapy ctDNA load was statistically significant and correlated with MTV (*p* = 0.0059)	Enrichment v3.0.0 and Variant Studio v3.0 For variant calling: Exome Varian, Server and ExAc	Illumina, San Diego, CA, USA	ctDNA	96 newly diagnosed pediatric patients enrolled in the EuroNet-PHL-C2 trial [23]	Use NGS on ctDNA from cHL pediatric patients	Desch et al., (2019) [24]
ctDNA mirrors genetic landscape of isolated HRS cells ctDNA may serve to personalize therapeutic decisions	Mutations identified in ctDNA and biopsies were highly concordant (87.50%) Treatment pressure induced differential patterns of clonal selection	BWA software and SAM tool. VarScan2 and Integrative Genome Viewer software (IGV)	Illumina, San Diego, CA, USA	FFPE tissue and ctDNA	80 cHL new diagnoses and 32 refractory patients	Identify the genetics of cHL in different clinical phases as well as its modifications on treatment	Spina et al., (2018) [25]
Drugs against members of JAK/STAT, NF-kB and BCR could be rationally used in cHL	Variants in EP300 (12.3%), CSFR2B (12.3%), BTK (10.5%) and STAT6 (10.5%). Frequent mutations involving the BCR pathway	Torrent Suite, Integrative Genomics Viewer (IGV), RAMSES, PROVEAN and Alamut algorithms	Ion Torrent^TM^, Thermo Fisher Scientific, NY, USA	FFPE tissue and cHL-derived cell lines	57 cHL samples and 6 cHL-derived cell lines	Describe the mutational landscape of cHL	Mata et al., (2017) [6]

NGS, next-generation sequencing; cHL, classic Hodgkin lymphoma; FL, follicular lymphoma; FFPE, formalin-fixed paraffin-embedded; ctDNA, circulating tumor DNA; PCR, polymerase chain reaction; MTV, metabolic tumor volume; CGP, comprehensive genomic profiling; HRS, Hodgkin and Reed–Sternberg; BCR, B cell receptor.

## Data Availability

Not applicable.

## Supplementary Materials

The following supporting information can be downloaded at: https://www.mdpi.com/article/10.3390/diagnostics12040963/s1. Table S1. List of genes included in the NGS panels of the documents analyzed in this systematic review. Table S2. Proposal of NGS panel for classic Hodgkin lymphoma.
