# Supplementary material for: The Need for Standardization in Next-Generation Sequencing Studies for Classic Hodgkin Lymphoma: A Systematic Review
Source: Diagnostics (Basel). 2022 Apr 12;12(4):963. doi: 10.3390/diagnostics12040963 (PMC9027849; doi:10.3390/diagnostics12040963)
Supplement: Supplementary file 1 [file diagnostics-12-00963-s001.zip › diagnostics-1658875-supplementary.pdf]

diagnostics

Supplementary Material

Systematic Review

The Need for Standardization in Next-Generation Sequencing Studies for Classic Hodgkin Lymphoma.

Antonio Santisteban-Espejo[1,2,3], Irene Bernal-Florindo[2,*], Jose Perez-Requena[1], Lidia Atienza-Cuevas[1], Julia Moran-Sanchez[3,4], María del Carmen Fernandez-Valle[4], Raquel Romero-Garcia[2] and Marcial Garcia-Rojo[1,2]

[1]Department of Pathology, Puerta del Mar University Hospital, 11009 Cadiz, Spain; antoniosantistebanespejo@gmail.com (A.S.E.); jose.perez.sspa@juntadeandalucia.es (J.P.-R.); lidia.atienza.sspa@juntadeandalucia.es (L.A.-C.); marcial.garcia.sspa@juntadeandalucia.es (M.G.-R.)

[2]Institute of Research and Innovation in Biomedical Sciences of the Province of Cadiz (INiBICA), 11009 Cadiz, Spain; raquel.romero.garcia@juntadeandalucia.es

[3]Department of Medicine, Faculty of Medicine, University of Cadiz, 11003 Cadiz, Spain; juliamorsan@gmail.com

[4]Department of Hematology and Hemotherapy, Puerta del Mar University Hospital, 11009 Cadiz, Spain; macafeva@hotmail.com

* Correspondence: iriberni@hotmail.com

Supplementary Table S1. List of genes included in the NGS panels of the documents analyzed in this systematic review.

Study [reference]	Genes included in the NGS panel	
Trecourt et al. [9]	ARID1A	ITPKB
	BCL2	KLF2
	BCOR	KMT2D/MLL2
	BIRC3	MEF2B
	BRAF	MFHAS1
	BTK	MYD88
	CARD11	NOTCH1
	CCND1	NOTCH2
	CD58	PIM1
	CD79A	PLCG2
	CD79b	PRDM1
	CDKN2A	PTPRD
	CDKN2B	RHOA
	CIITA	SF3B1
	CREBBP	SOCS1
	CXCR4	STAT3
	EP300	STAT6
	EZH2	TCF3
	FBXW7	TET2
	FOXO1	TNFAIP3
	GNA13	TRAF2
	ID3	TRAF3
	IDH2	XPO1
	IRF4	
Alcoceba et al. [1]	ARID1A	KMT2D
	ATM	LRRN3
	B2M	MYC
	BIRC3	NFKBIE
	BTG1	NFKBI1A
	BTK	P2RY8
	CARD11	PCBP1
	CD36	PIK3CA
	CIITA	PIM1
	CREBBP	PTPN1
	CSF2RB	SOCS1
	CXCR4	SPEN
	EP300	STAT6
	EZH2	TBL1XR1
	FBXW7	TNFAIP3
	GNA13	TNFRSF14
	HIST1H1C	TP53
	HIST1H1E	XPO1
	IGLL5	ZMYM3
	IKBKB	MYD88
	ITPKB	CCND3
Camus et al. [6]	NFKBIE	

	ITPKB<br>PTPN1<br>TNFAIP3<br>SOCS1<br>STAT6<br>B2M<br>XPO1<br>GNA13	
Mata et al. [10]	ABL1<br>B2M<br>BCL10<br>BTK<br>CARD11<br>CASP8<br>CD19<br>CD38<br>CREBBP<br>CSF1R<br>CSF2<br>CSF2RB<br>CYLD<br>EP300<br>FAS<br>IL32<br>LCP1<br>MYB	MYC<br>NFKBIA<br>NFKBIE<br>NOTCH1<br>NUMA1<br>PIK3CD<br>PTPN1<br>REL<br>RET<br>SH3BP5<br>SMARCA4<br>SOCS1<br>STAT3<br>STAT6<br>TNFAIP3<br>TP53<br>TRAF3
Liang et al. [11]	ABL1<br>BRIP1<br>CHEK2<br>FANCA<br>GNAQ<br>MAP2K1<br>NFKBIA<br>PPP2R1A<br>SMARCD1<br>AKT1<br>BTG1<br>CHUK<br>FANCC<br>GNAS<br>MAP2K2<br>NKX2-1<br>PRDM1<br>SMO<br>AKT2<br>BTK<br>CIC<br>FANCD2<br>GPR124<br>MAP2K4<br>NOTCH1	ATRX<br>CDK12<br>DNMT3A<br>FGF3<br>IRF4<br>MPL<br>PARP1<br>RARA<br>TNFAIP3<br>AURKA<br>CDK4 D<br>OT1L<br>FGF4<br>IRS2<br>MRE11A<br>PARP2<br>RB1<br>TNFRSF14<br>AURKB<br>CDK6<br>EGFR<br>FGF6<br>JAK1<br>MSH2<br>PARP3

	PRKAR1A	REL
	SOCS1	TOP1
	AKT3	AXL
	C17orf39	CDK8
	CRBN	EMSY
	FANCE	FGF7
	GSK3B	JAK2
	MAP3K1	MSH6
	NOTCH2	PARP4
	PRKDC	RET
	SOX10	TP53
	ALK	BACH1
	CARD11	GRIN2A
	CREBBP	EP300
	FANCF	FGFR1
	HGF	JAK3
	MAP3K13	MTOR
	NOTCH3	PAX5
	PRSS8	RICTOR
	SOX2	TRRAP
	ALOX12B	BAP1
	CASP8	SPOP
	CRKL	EPHA3
	FANCG	FGFR2
	HLA-A	JUN
	MCL1	MUTYH
	NOTCH4	PDGFRA
	PTCH1	RNF43
	SPEN	TSC1
	APC	BARD1
	CBFB	SETD2
	CRLF2	EPHA5
	FANCI	FGFR3
	HRAS	KDM5A
	MDM2	MYC
	NPM1	PDGFRB
	PTEN	RPA1
	SRC	TSC2
	APCDD1	BCL2
	CBL	PBRM1
	CSF1R	EPHB1
	FANCL	FGFR4
	IDH1	KDM5C
	MDM4	MYCL1
	NRAS	PDK1
	PTPN11	RPTOR
	STAG2	TSHR
	AR	BCL2L2
	CCND1	FOXL2
	CTCF	ERBB2
	FANCM	FLT1

	IDH2	KDM6A
	MED12	MYCN
	NSD1	PIK3C2G
	RAD50	RUNX1
	STAT4	VHL
	ARAF	BCL6
	CCND2	CTNNA1
	CTNNB1	ERBB3
	FAT3	FLT3
	IGF1	KDR
	MEF2B	MYD88
	NTRK1	PIK3C3
	RAD51	RUNX1T1
	STK11	WISP3
	ARFRP1	BCOR
	CCND3	CDKN1B
	CUL4A	ERBB4
	FBXW7	FLT4
	IGF1R	KEAP1
	MEN1	MYST3
	NTRK2	PIK3CA
	RAD51B	SF3B1
	SUFU	WT1
	ARID1A	BCORL1
	CCNE1	CDKN2A
	CUL4B	ERG
	FGF10	GATA1
	IGF2	KIT
	MET	NBN
	NTRK3	PIK3CG
	RAD51C	SH2B3
	SYK	XPO1
	ARID2	BLM
	CD79A	CDKN2B
	CYP17A1	ESR1
	FGF12	GATA2
	IKBKE	KLHL6
	MITF	NCOR1
	NUP93	PIK3R1
	RAD51D	SMAD2
	TBX3	XRCC3
	ASXL1	BRAF
	CD79B	CDKN2C
	DAXX	EZH2
	FGF14	GATA3
	IKZF1	KRAS
	MLH1	NF1
	PAK3	PIK3R2
	RAD52	SMAD4
	TET2	ZNF217
	ATM	BRCA1

	CDC73	CEBPA
	DDR2	FAM123B (WTX)
	FGF19	GNA11
	IL7R	LMO1
	MLL	NF2
	PAK7	PMS2
	RAD54L	SMARCA4
	TGFBR2	ZNF703
	ATR	BRCA2
	CDH1	CHEK1
	DIS3	FAM46C
	FGF23	GNA13
	INHBA	LRP1B
	MLL2	NFE2L2
	PALB2	PNRC1
	RAF1	SMARCB1
	TIPARP	
Desch et al. [7]	ACTG1	MEF2B
	CSF2RB	MYC
	ARID1A	NCOR2
	FOXO1	NSD2
	ATM	PIM1
	IGLL5	POT1
	ATP6AP1	POU2F2
	ITPR3	PRDM1
	ATP6V1B2	PTEN
	MYB	PTPN11
	B2M	RB1
	NFKBIA	S1PR2
	BCL2	SALL3
	NFKBIE	SAMHD1
	BCL6	SGK1
	NUP214	SMARCA4
	BIRC3	SOCS1
	PTPN1	SPEN
	BTG1	TBL1XR1
	TET2	TCF3
	CARD11	TMEM30A
	CCND1	TNF
	CCND3	TNFAIP3
	CCT6B	TNFRSF14
	CD58	TP53
	CD70	TRAF3
	CD83	BRAF
	CDH2	BTK
	CDK4	CD79B
	CDKN2A	CXCR4
	CREBBP	IDH1
	CYLD	PLCG2
	DDX3X	KRAS
	DIS3	CIITA

	**DUSP2**	MYD88
	EP300	NOTCH1
	ETS1	NRAS
	EXOSC1	SF3B1
	EZH2	STAT6
	EZR	XPO1
	FAM46C	**Genomic**
	FBXW7	**regions for**
	FGFR3	**structural**
	GNA13	**variants**
	HIST1H1E	BCL1-MTC all
	HIST1H1C	IgH-VH
	HIST1H1D	BCL2-3MBR
	HIST1H3B	BCL2-5mcr
	HLA-A	BCL2-MBR
	ID3	BCL2-mcr
	INTS12	BCL6-5΄UTR
	IRF4	CD274
	IRF8	CPA5
	ITPKB	IRF5
	KLF2	all IgH-DH
	KLHL6	all IgH-JH
	KMT2C	all IgH-switch
	KMT2D	regions
	LTB	all IgK-JK
	MAP2K1	all IgL-JL
	MED12	LEP
		MIR15A
		MIR16-1
		REL
		MYC
	ARID1A	KLF2
	ATM	KLHL6
	B2M	KMT2D
	BCL2	KRAS
	BCL6	MAP2K1
	BCOR	MAP3K14
	BIRC3	MEF2B
	BRAF	MYC
	BTG	MYD88
	BTK	NFKBIE
Spina et al. [8]	CARD11	NOTCH1_3'UTR
	CCND1	NOTCH1_EX34
	CCND2	NOTCH2_EX34
	CCND3	NRAS
	CD36	P2RY8
	CD58	PCBP1
	CD70	PIK3CA
	CD79A	PIM1
	CD79B	PLCG2
	CIITA	POT1

	CREBBP	PRDM1
	CXCR4	RIPK1
	EGR2	RRAGC
	EP300	S1PR2
	EZH2	SIN3A
	FBXO11	SPEN
	FBXW7	STAT3
	FGFR2	STAT6
	FOXO1	TBL1XR1
	GNA13	TCF3
	HIST1H1C	TET2
	HIST1H1E	TNFAIP3
	HIST1H3B	TNFRSF14
	ID3	TP53
	IKBKB	TRAF2
	IRAK1	TRAF3
	IRF4	WHSC1
	IRF8	XPO1
	ITPKB	ZMYM3
Mata et al. [5]	B2M	TNFRSF14
	CARD11	CSF2RB
	CASP8	FAS
	CSF1R	LCP1
	BCL10	LTB
	NFKBIA	MAPK3
	STAT6	MDM2
	BTK	NUMA1
	IL32	PLCG2
	MYB	SMARCA4
	SH3BP5	NOTCH1
	ABL1	CREBBP
	ADAM8	EP300
	CD19	STAT3
	CD38	MYC
	NFKB2	EZH2
	PIK3CD	MYD88
	RET	

Supplementary Table S2. Proposal of NGS panel for classic Hodgkin lymphoma.

Gene	Chromosome	Number of Amplicons	Total bases	Covered bases	Missed bases	Coverage
ID3	Chr1	5	400	400	0	1.000
ARID1A	Chr1	87	7258	7187	71	0.969
BCL10	Chr1	10	762	762	0	1.000
NOTCH2	Chr1	102	8149	7958	191	0.731
XPO1	Chr2	51	3696	3674	22	0.916
CXCR4	Chr2	13	1126	1126	0	1.000
SF3B1	Chr2	68	4505	4478	27	0.961
CASP8	Chr2	24	1913	1889	24	0.984
MYD88	Chr3	14	1054	1054	0	1.000
CD38	Chr4	15	1063	1063	0	1.000
CSF2	Chr5	9	515	515	0	1.000
CSF1R	Chr5	51	3339	3339	0	1.000
NFKBIE	Chr6	21	1623	1623	0	1.000
MYB	Chr6	38	2606	2596	10	0.984
TNFAIP3	Chr6	26	2533	2515	18	0.990
CARD11	Chr7	54	3945	3945	0	1.000
BRAF	Chr7	42	2661	2655	6	0.997
EZH2	Chr7	40	2636	2636	0	1.000
MYC	Chr8	15	1425	1425	0	1.000
PTPRD	Chr9	84	6539	6516	23	0.996
CDKN2A	Chr9	11	1012	1008	4	0.996
ABL1	Chr9	45	3769	3759	10	0.991
NOTCH1	Chr9	111	8348	8274	74	0.973
TRAF2	Chr9	21	1706	1706	0	1.000
FAS	Chr10	17	1188	1188	0	1.000
CCND1	Chr11	14	988	988	0	1.000
BIRC3	Chr11	28	1975	1971	4	0.997
STAT6	Chr12	42	2964	2955	9	0.996
FOXO1	Chr13	19	2608	1929	79	0.939
LCP1	Chr13	30	2184	2184	0	1.000
NFKBIA	Chr14	16	1074	1074	0	1.000
B2M	Chr15	7	420	420	0	1.000
IL32	Chr16	9	805	605	200	0.896
CREBBP	Chr16	95	7949	7926	23	0.995
CD19	Chr16	32	1954	1954	0	1.000
CYLD	Chr16	43	3211	3144	67	0.978
PLCG2	Chr16	62	4438	4438	0	1.000
TP53	Chr17	24	1503	1503	0	1.000
CD79B	Chr17	12	813	813	0	1.000
BCL2	Chr18	9	793	793	0	1.000
TCF3	Chr19	35	2572	2567	5	0.997
KLF2	Chr19	12	1128	968	160	0.936
**Gene**	**Chromosome**	**Amplicons**	**Total bases**	**Covered bases**	**Missed bases**	**Coverage**

MEF2B	Chr19	16	1267	1267	0	1.000
CD79A	Chr19	12	781	781	0	1.000
CSF2RB	Chr22	38	2954	2954	0	1.000
EP300	Chr22	97	7865	7865	0	1.000
BTK	ChrX	33	2462	2402	0	1.000
